# Development of Advanced Solid-State Thermochromic Materials for Responsive Smart Window Applications

**DOI:** 10.3390/polym16162385

**Published:** 2024-08-22

**Authors:** Kai Zeng, Chang Xue, Jinbo Wu, Weijia Wen

**Affiliations:** 1 Materials Genome Institute, Shanghai University, Shanghai 200444, China; zengkai-mgi@shu.edu.cn (K.Z.); jinbowu@t.shu.edu.cn (J.W.); 2 Faculty of Materials Science, Shenzhen MSU-BIT University, Shenzhen 518115, China; 3 Department of Physics, The Hong Kong University of Science and Technology, Clear Water Bay, Kowloon, Hong Kong

**Keywords:** smart materials, emulsion polymerization, solid-state thermochromic materials, transmittance, thermal management, environmental sustainability

## Abstract

This study introduces the synthesis and detailed characterization of a novel thermochromic material capable of reversible alterations in its thermotropic transmittance. Through an emulsion polymerization process, this newly developed material is composed of 75–85% octadecyl acrylate and 0–7% allyl methacrylate, demonstrating a pronounced discoloration effect across a narrow yet critical temperature range of 24.5–39 °C. The synthesized powder underwent a battery of tests, including differential scanning calorimetry and thermogravimetric analysis, as well as scanning electron microscopy. These comprehensive evaluations confirmed the material’s exceptional thermal stability, uniform particle size distribution, and strong anchoring properties. Building upon these findings, we advanced the development of thermochromic polyvinyl butyral films and laminated glass products. By utilizing a coextrusion technique, we integrated these films into laminated glass, setting a new benchmark against existing glass technologies. Remarkably, the incorporation of thermochromic PVB films into laminated glass led to a significant reduction in solar irradiance of 20–30%, outperforming traditional double silver low-emissivity glass. This achievement demonstrates the exceptional shading and thermal insulation properties of the material. The research presented herein not only pioneers a valuable methodology for the engineering of smart materials with tunable thermotropic transmittance but also holds the key to unlocking enhanced energy efficiency across a spectrum of applications. The potential impact of this innovation on the realm of sustainable building materials is profound, promising significant strides toward energy conservation and environmental stewardship.

## 1. Introduction

In the field of construction, energy utilization has emerged as a critical issue, with a staggering 40% of total energy consumption in regions such as the European Union, the United States, and other developed nations attributed to this sector [1]. In China, the architectural sector’s energy consumption represented 19.1% of the country’s total energy use in 2012, a figure that increased at an average annual growth rate of approximately 8.3% from 2001 to 2012 [2]. In response, researchers have explored ways to reduce energy consumption and improve conservation within the architectural domain worldwide. A variety of materials and energy management strategies have been developed, with the integration of structural and functional elements into material design leading to significant breakthroughs in solar energy management within buildings. These innovations are instrumental in reducing carbon emissions and bolstering the energy efficiency of buildings, all in the pursuit of a more sustainable future [3,4,5].

Scientists have harnessed a diverse array of methods to reduce energy usage in building operations. These strategies include the application of lightweight porous materials [6], thermal insulation coatings [7], solar reflective coatings [8], and cool light-color paints. Moreover, the use of solar spectral selective absorbing coatings [9] and the strategic placement of ventilation windows on rooftops are among the tactics employed to enhance thermal insulation. Despite the range of available methods, there are inherent limitations in fully exploiting solar energy throughout the seasons, which impacts the ability to maintain optimal warmth in winter and coolness in summer. Nonetheless, the advent of energy-efficient windows made from glass combined with color-changing materials offers an auspicious solution. These chromogenic materials adjust their optical properties in response to external stimuli, presenting a dynamic thermal regulation approach. Classified according to their stimuli, photochromic [10], electrochromic [11], and thermochromic [12] materials react to changes in light [13], electric fields [14], and temperature [15], respectively.

Electrochromic glass is particularly notable as a leading innovation in smart glass technology and is capable of reversible transitions between transparent and tinted states upon the application of a specific voltage. In its tinted state, it markedly diminishes visible light transmittance and the solar heat gain coefficient but retains transparency, making it an effective tool for light and heat management within buildings [16]. However, electrochromic glass has shortcomings, such as its inability to effectively reduce glare from direct sunlight, especially intense glare from east–west orientations or top surfaces during periods of strong outdoor illumination or direct sun exposure [17]. Thermochromic materials, on the other hand, are capable of altering their color or optical transmission in response to temperature changes [12,18,19]. As the surface temperature of thermochromic materials varies, so do their optical and thermal properties [20]. At the transition temperature, these materials undergo a property change. Their defining characteristic is their sensitivity to surface temperature: above the transition temperature, they absorb and reflect most of the sun’s infrared rays while partially absorbing and scattering visible light.

Conversely, when the surface temperature is below the threshold, sunlight can pass directly through the material into the room. Given the desired performance characteristics, intrinsic modification of glass presents a challenge. The pure thermochromic material, VO_2_ film, undergoes a phase transition from a metal to a semiconductor at a critical temperature of 68 °C, reflecting the near-infrared region with high efficiency at temperatures above this point [21]. To increase the practicality and industrial viability of VO_2_ films, researchers have delved into the realm of doping, specifically incorporating metal ions such as tungsten (W) into the VO_2_ lattice. This innovative approach is designed to lower the phase transition temperature of VO_2_ films, making them more suitable for a broader range of everyday applications [22]. The doping process not only adjusts the temperature at which VO_2_ transitions between phases but also reduces the energy threshold required for this transition, enabling it to occur at lower temperatures. For example, the introduction of only 0.4% tungsten can significantly lower the lattice transition energy from 15.2 °C to 8.3 °C, effectively reducing the phase transition temperature from 68 °C to 56 °C [23].

Chen et al. [24] developed a sophisticated bilayer VO_2_ film, which is only 55 nanometers thick and includes both a VO_2_ layer and a titanium dioxide antireflective layer. This groundbreaking structure led to a substantial increase in the film’s visible light transmittance at lower temperatures, with a notable increase from 40.3% to 61.5% at 20 °C. Shen et al. [25] achieved a uniform dispersion of zirconium-doped VO_2_ nanoparticles within an oily polyurethane solution, which, when applied to a polyethylene terephthalate substrate and dried, resulted in the successful formation of a composite film [26]. This VO_2_-PU composite film displays a visible light transmittance (τvis) of 60.4% and impressive solar modulation ability, as indicated by a solar transmittance change (Δτsolar) of 14.1% at 25 °C [27]. Long et al. [28] have also made progress in this field, developing a modified composite film with a phase transition temperature of 41.3 °C for application on the exterior surface of hollow glass. Outdoor testing facility trials have demonstrated that this modified VO_2_ film can reduce the cooling energy consumption by 11.1% compared with that of standard hollow glass.

Polymer thermochromic films represent a distinct category of thermochromic films, achieving thermochromism through the integration of specific ligand metal ions, such as nickel or cobalt, into a transparent polymer matrix, such as polyvinyl butyral (PVB). These metal ions undergo color changes at various temperatures due to their ligand structures, thus enabling thermochromic properties and reversible transmittance changes across the visible and near-infrared spectra [29]. Giovannini et al. [30] and Lu et al. [31] utilized building simulation techniques to integrate visible light and near-infrared data from ligand metal ion-based thermochromic films at various temperatures into a building model. Their study revealed that employing such thermochromic glass could lead to energy savings of 3% to 10% compared with traditional glass under identical continental climate conditions, with additional potential to reduce lighting energy usage by 5% to 20%.

In a pivotal study by Lee [32], the focus was on the synthesis of reversible thermotropic materials via polymerization techniques, resulting in a novel reactive thermotropic liquid crystalline polymer. El-Deeb [33] explored characterization techniques for thermochromic materials, employing UV–Vis and Raman spectroscopy to examine changes in optical properties with temperature, whereas SEM and DSC microscopy techniques were used to investigate the microstructure and phase transitions of the materials. Busi Kumar Babu et al. [34] addressed the applications of reversible smart materials in smart windows, highlighting their ability to actively modulate light and heat transmission, thereby improving energy efficiency and indoor comfort levels and highlighting their potential environmental benefits, such as reduced carbon emissions.

In this novel study, we successfully synthesized an advanced solid-state thermochromic powder, which is highly important for the development of thermochromic materials. By integrating this powder into transparent resins, we have significantly bolstered the material’s resilience and dimensional integrity, culminating in a more robust end product. The resulting smart film not only inherits the dynamic thermochromic properties of the powder but also actively regulates solar transmittance. At temperatures above a predefined threshold, the film augments solar scattering to curtail heat absorption, whereas below this threshold, it permits increased solar penetration. This research is not only a testament to our commitment to scientific advancement but also a critical step toward sustainable construction practices, offering newly developed solutions to the pressing issue of energy conservation.

## 2. Synthesis and Characterization

### 2.1. Synthesis

We commenced the synthesis by meticulously preparing the aqueous phase, which was meticulously maintained at 35 °C and composed of 0.30 g of sodium dodecyl sulfate, 0.075 g of NaHCO_3_, and 140 g of deionized water. Concurrently, under well-ventilated conditions, 75 wt% ODA completely melted at 60 °C. The monomer blend, consisting of 10 wt% butyl acrylate, 5 wt% allyl methacrylate (ALMA), and 10 wt% styrene, was prewarmed in a water bath at 35 °C before being combined with the molten ODA under continuous magnetic stirring. This mixture was then held at 35 °C for an additional 15 min to ensure thorough blending.

In the next phase, 0.6 g of the oil-soluble initiator Dilauroyl peroxide was introduced into the monomer blend, ensuring complete dissolution through magnetic stirring. The blend was subsequently stirred for 10 min at 35 °C to achieve a preliminary dispersion, forming an o/w crude emulsion. This emulsion was then subjected to ultrasonic homogenization for 30 min, creating an o/w microemulsion while being cooled in a 25 °C water bath with strict internal temperature monitoring.

The o/w microemulsion was degassed under an argon atmosphere with stirring for 15 min, after which the inert gas flow was carefully reduced, and the mixture was heated to 70 °C. Polymerization was allowed to proceed at this temperature with consistent stirring for 120 min.

**Figure 1:**
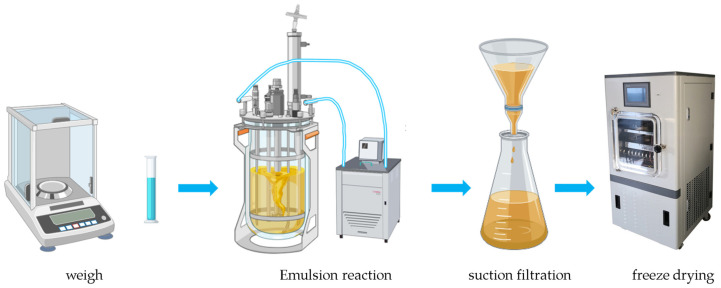
Fabrication process of thermochromic powder.

A mixed phase consisting of a monomer phase with 96 wt% methyl methacrylate and 4 wt% ethyl acrylate and an aqueous phase with 0.020 g sodium dodecyl sulfate, 0.010 g NaHCO_3_, 0.020 g NaPDS, and 20 g deionized water was subsequently stirred vigorously into the o/w microemulsion for 30 min. The oil bath was then removed, and the particles were dispersed and solidified with 150 mL of a 0.5% magnesium sulfate solution under vigorous agitation. The solid product was vacuum-filtered, followed by five thorough washes with 250 mL of water each to eliminate any water-soluble polymeric substances, the fabrication process is shown in Figure 1.

The thermochromic PVB film was ultimately produced via a twin-screw extruder. This process involved blending 90 parts of PVB, 10 parts of the synthesized thermochromic powder, and 33 parts of the plasticizer DBP, which were then extruded to form the final PVB film.

### 2.2. Characterization

The surface morphology and microstructure of the synthesized materials were examined via a JEOL FE–SEM 6700F scanning electron microscope from Akishima, Japan. Differential scanning calorimetry (DSC) and thermogravimetric (TG) measurements were conducted with a PerkinElmer DSC-7 instrument from the Waltham, MA, USA, using samples weighing 2.0–5.0 mg placed in aluminum pans. The DSC scans were performed at a rate of 2 °C per minute.

The fluorescence characteristics of the materials were determined via a Hitachi F-4500 fluorescence spectrophotometer from Tokyo, Japan. The optical properties of the thermochromic laminated glass were monitored over a wavelength range of 300–2500 nm via a Lambda 1050 spectrophotometer from PerkinElmer, Waltham, MA, USA equipped with a heating unit. Outdoor comparative tests assessing temperature, illumination, and irradiance were conducted via a state-of-the-art online testing device, ensuring accurate and real-time data acquisition.

### 2.3. Outdoor Experimental 

The experimental setup featured a downsized physical model of a testing chamber, expertly crafted from 75 mm-thick polystyrene foam encased in color steel sandwich panels. The testing chamber was meticulously designed at a 1:4 scale. With dimensions of 1.2 m in length, 1.1 m in width, and 0.8 m in height, the chamber was equipped with an external window measuring 0.6 m by 0.5 m. The aspect ratio of the windows to the walls was carefully set at 0.34, and the windows included a sill positioned at a height of 0.2 m.

To counteract the effects of high summer temperatures on the thermal environment and the experimental equipment within the testing chamber, a protective enclosure, measuring 2.0 m × 1.9 m × 1.5 m, was strategically installed around the chamber. This configuration was designed to simulate an actual office room environment, similar to one with an air conditioning system operating from an adjacent room during the summer months.

Temperature monitoring within the testing chamber was meticulously carried out via T-type thermocouples, which were tasked with measuring both the indoor temperature and the surface temperatures of the glass—both on the interior and exterior sides. A state-of-the-art temperature data collector was used to automatically gather and log these readings at 10 min intervals.

Illuminance levels within and outside the testing chambers were accurately measured with an illuminance sensor, and in accordance with the temperature data collection, these readings were automatically recorded every 10 min via a data collector. Moreover, a solar radiation sensor was deployed to measure the intensity of the indoor solar radiation and the total solar radiation striking the vertical wall of the test chamber. These radiation data were also systematically collected and recorded at 10 min intervals.

## 3. Results

### 3.1. Influence of ODA on the Temperature and Transmittance Characteristics of Thermochromic Materials

The exploration of microemulsion polymerization with organic monomers has captured significant interest across the globe. In our study, ODA was strategically selected as the polymerization matrix for incorporating thermotropic functional groups. This choice led to the successful synthesis of a polymer shell microemulsion with a particle size of approximately 5 μm through emulsion polymerization. The integration of ODA monomers significantly enhances the thermochromic polymer’s capacity to modulate the transmittance of PVB in response to temperature variations. However, the inclusion of ODA in excess quantities may result in the increased formation of homopolymers in the aqueous phase, potentially compromising the overall transmittance of the material. With increasing ODA content, there was a notable increase in system viscosity and a concomitant increase in latex particle size. When modified with PVB, the transmittance at room temperature initially increases but then decreases, mirroring a similar trend at higher temperatures.

**Figure 2:**
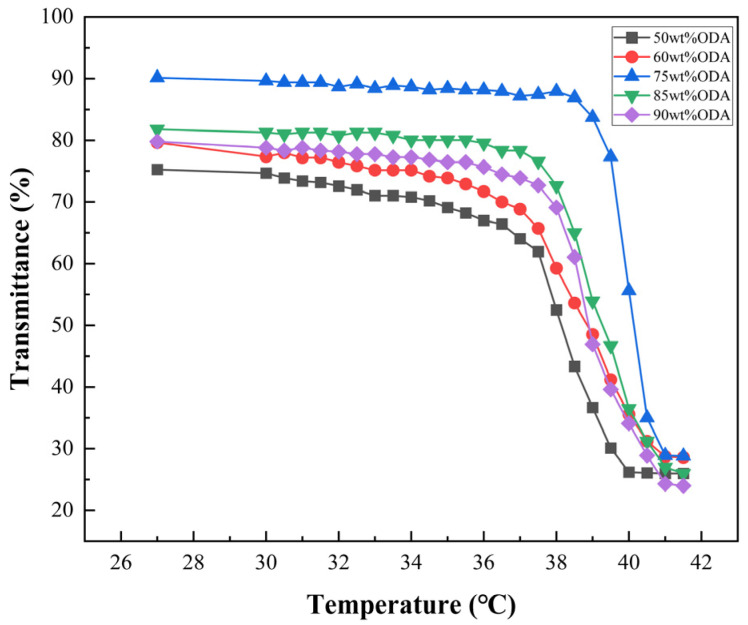
Visible light transmittance of laminated glass prepared with thermochromic powder synthesized with different concentrations of ODA monomers at different temperatures.

Figure 2 elegantly depicts the relationship between visible light transmittance (τvis) and the phase transition temperature of laminated glass containing ODA monomers and thermochromic dimming materials.

The data illustrate that increasing the ODA concentration from 75 wt% to 85 wt% marginally decreases the phase transition temperature (Ts) from 40.2 °C to 39.5 °C. Nonetheless, further increasing the ODA content from 85 wt% to 90 wt% led to a minimal reduction in the phase transition temperature of approximately 1 °C. This finding indicates that beyond a certain concentration threshold, increasing the proportion of ODA monomers has a negligible effect on diminishing the phase transition temperature of the thermochromic material.

### 3.2. Effect of the ALMA Monomer on the Temperature and Transmittance Characteristics of Thermochromic Materials

**Figure 3:**
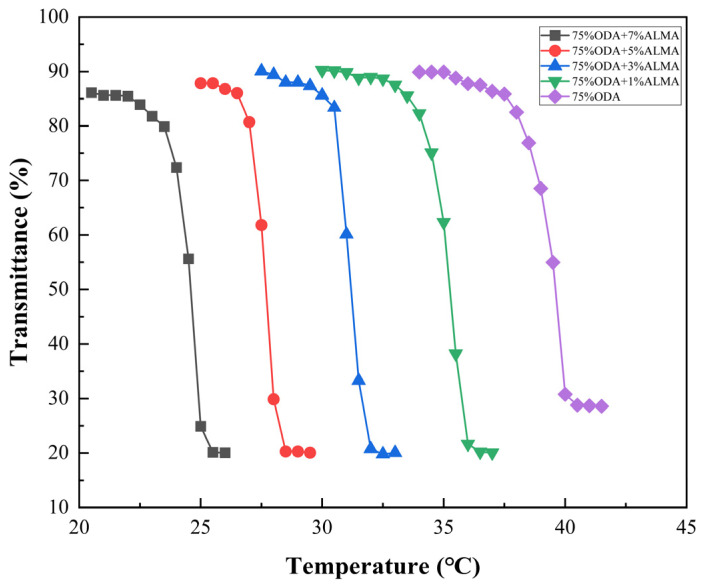
Visible light transmittance of laminated glass prepared with thermochromic powder synthesized at different concentrations of 75% ODA monomer with various ALMA concentrations at different temperatures.

At an ODA monomer concentration of 75%, the phase transition temperature of the thermochromic powder can be finely adjusted from 24.5 °C to 39.5 °C by varying the ALMA content. Figure 3 clearly shows the relationship between the visible light transmittance (τvis) of laminated glass, which incorporates a thermochromic powder with a 75% ODA formulation, and various concentrations of ALMA. An increase in ALMA from 0 to 7 wt% substantially decreases the phase transition temperature (Ts) from 39.5 °C to 24.5 °C. This pronounced reduction in Ts is likely due to the structural expansion of the thermochromic powder induced by the presence of ALMA in the branched chains, which weakens the bonding forces between the ODA polymer and polyethylene adipate, facilitating the aggregation and precipitation of the polymer at lower temperatures.

However, further increasing the ALMA content beyond the optimal level is advised. The excessive incorporation of ALMA can cause the polymer to exhibit turbidity at or below room temperature (25 °C), potentially leading to an undesirable color shift in practical applications and adversely affecting product performance, especially in window applications where clarity is paramount.

This provides a visual representation of the visible light transmittance of laminated glass prepared with thermochromic powder synthesized at various concentrations of 75% ODA monomer alongside different ALMA concentrations at various temperatures.

### 3.3. SEM

**Figure 4:**
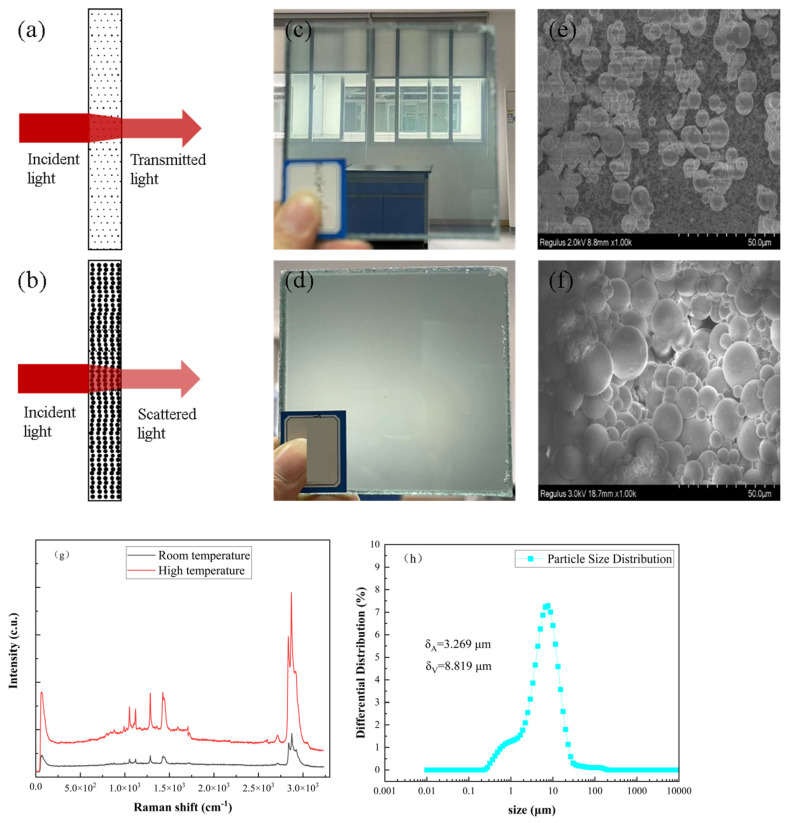
Synthesized thermochromic powder and PVB film for light absorption: (**a**) room temperature; (**b**) high temperature; (**c**,**d**) visibility through a polymer at room temperature; (**e**,**f**) optical micrographs of the same polymers at different temperatures; (**g**) Raman scattering spectra of the synthesized thermochromic powder; (**h**) particle size distribution of the solid-state thermochromic powder: δ_A_: average particle size of the surface area; δV: volume average particle size.

Figure 4 presents a scanning electron microscope (SEM) image of a sample synthesized with a composition of 75% ODA and 1% ALMA. The image reveals a uniform array of spherical particles that are clearly free from significant agglomeration and feature a rugged surface marked by pronounced anchor-like structures. These uniformly distributed surface protrusions are anticipated to effectively interlock with PVB, thus fostering the formation of an enhanced PVB film during the twin-screw melt extrusion process.

SEM analysis further provides precise measurements, indicating that the surface area mean diameter of the solid-state thermochromic powder is 3.269 μm, with a volume mean diameter of 8.819 μm. An increase in particle size, which correlates with a larger surface area, may increase the light absorption capacity of thermochromic materials, potentially intensifying the thermochromic effect as the particles absorb more heat. However, larger particle sizes could also impact thermal conductivity, resulting in increased size, larger gaps, and extended pathways for heat transfer, which might lead to slower responses to temperature fluctuations and diminished stability in terms of the thermochromic effect. Moreover, larger particles can introduce haze during processing, adversely affecting the clarity and performance of the final product.

From the Raman spectroscopy analysis conducted at 25 °C and 50 °C, the characteristic peaks at 90.89 cm^−1^, 1230 cm^−1^, 1450 cm^−1^, and 2750 cm^−1^ remained consistent in position, indicating that no inherent material changed across the various temperatures. However, the heightened amplitude of these peaks at 50 °C suggests temperature-induced alterations in the material’s structure or order, changes that could be linked to the thermochromic properties as such effects are typically the consequence of structural transformations within the material. When subjected to heat, modifications in the molecular or lattice structure can precipitate changes in optical properties, often reflected in parameters such as the band structure and scalar coupling.

The intensity of the Raman shift peaks also acts as an indicator of the excitation level and, to a degree, the quantity of molecules present. At elevated temperatures, an increased number of molecules are excited by the laser, corroborating the influence of the DSC characteristics on the material’s thermal variation. This interplay demonstrates the complex relationships among material structure, thermal properties, and their collective role in shaping the thermochromic behavior of a material.

### 3.4. TG–DSC

**Figure 5:**
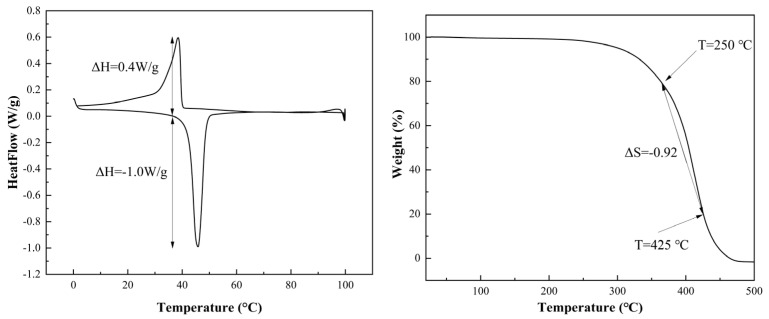
DSC and TG analysis of the synthesized thermochromic powder.

The experiments were conducted at a heating rate of 2 °C/min, and the results depicted in Figure 5 shed light on the thermal stability of the solid-state thermochromic powder. TG analysis revealed the onset of weight loss at 250 °C, with a significant reduction to 90% at 450 °C, suggesting that the material underwent a phase transition that initiated weight loss or decomposition at 250 °C. Additionally, the TG curve points to a 20% weight loss at 360 °C and 80% weight loss at 425 °C, implying potential intermediate phase transitions within this temperature range, contributing to the further decomposition of the material.

The DSC curve revealed noticeable heat flow changes at 40 °C during the heating cycle, with a pronounced heat absorption peak of −1.0 w/g at 42 °C. This peak likely signifies a phase transition occurring between 40 °C and 42 °C. Similarly, during the cooling cycle, the heat flow begins to change at 40 °C, reaching a peak release of 0.6 w/g at 39 °C. These heat flow variations are indicative of exothermic and endothermic transitions associated with structural and compositional changes within the thermochromic material.

These phase transitions, which are evident in both TG and DSC analyses, reflect modifications to the material’s structure, composition, and crystallography. Such transformations are crucial for adjusting the crystal lattice, which subsequently influences the optical properties of the material and induces a thermochromic effect—a reversible color change with temperature. Understanding these thermal behaviors is essential for refining the thermal response of thermochromic materials for diverse applications. The synthesized thermochromic powder material, which contains hydroxyl and carboxyl groups, engages in weak physical hydrogen bonding with the hydroxyl groups in the PVB molecules. An increase in the quality of the core-shell structure and an alteration in the material’s thermal transition properties are realized via the addition of methyl methacrylate, which creates protrusions that marginally lower the film’s transition temperature below 42 °C, as indicated by the DSC curves.

### 3.5. FTIR

**Figure 6:**
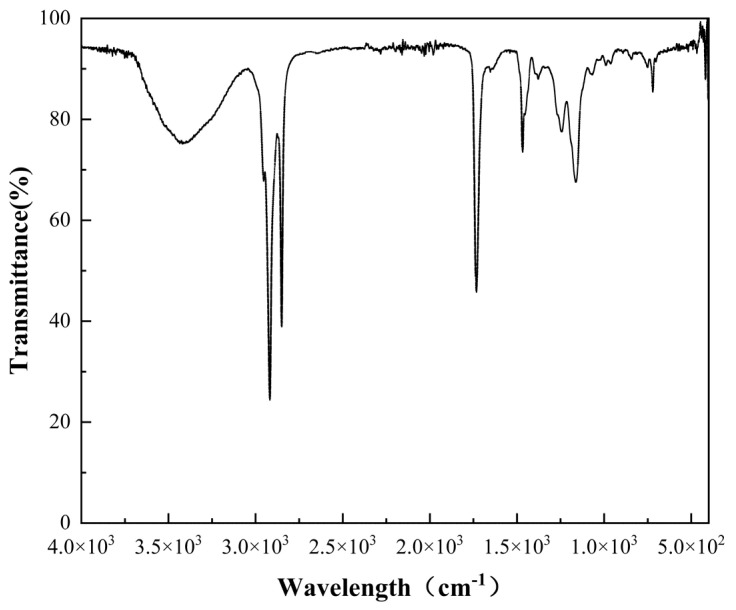
FTIR analysis of the synthesized thermochromic powder.

Figure 6 shows the FTIR (Fourier transform infrared) spectra of the synthesized thermochromic powder, revealing essential information about the molecular structure and its influence on the thermal properties. Within the spectrum, a broad peak indicative of multimolecular O–H associations is observed in the range of 3300–3600 cm^−1^. The C-H stretching vibrations are represented by a distinct peak in the 2800–2900 cm^−1^ region, and the presence of a carbon–carbon double bond is denoted by a peak within 1620–1680 cm^−1^.

The molecular structure is a critical determinant of the thermal performance and phase transition behavior of materials. For the synthesized thermochromic powders, the inclusion of short alkyl side chains elevates the decomposition temperature, suggesting a more thermally robust material. These side chains contribute to enhanced molecular mobility, which is associated with a reduced phase transition temperature, thereby facilitating a more pronounced thermochromic effect at lower temperatures and improving the material’s overall thermal stability.

Additionally, the incorporation of aromatic rings during synthesis enhances this thermal stability. Through π-electron delocalization, aromatic rings confer extra rigidity to the molecular structure, limiting rotational motion, increasing the melting point of the material, and increasing its thermal stability.

The incorporation of polar functional groups such as hydroxyl (–OH) or carbonyl (C=O) groups is also evident in the FTIR spectra. These groups promote stronger hydrogen bonding and dipole–dipole interactions, which influence the thermal behavior of the material. These interactions are not only vital for the material’s structural integrity but also play a significant role in defining the strength and nature of the thermochromic response.

In summary, the FTIR spectra provide insight into the complex interplay of functional groups in the thermochromic powder. The presence and arrangement of these groups within the material’s molecular architecture not only modify its inherent properties but also dictate the strength and mechanisms of the thermochromic transitions. Understanding these spectral signatures is key to tailoring the material for specific thermal responses and stability requirements in practical applications.

### 3.6. Mechanical Properties of the PVB Film

**Figure 7:**
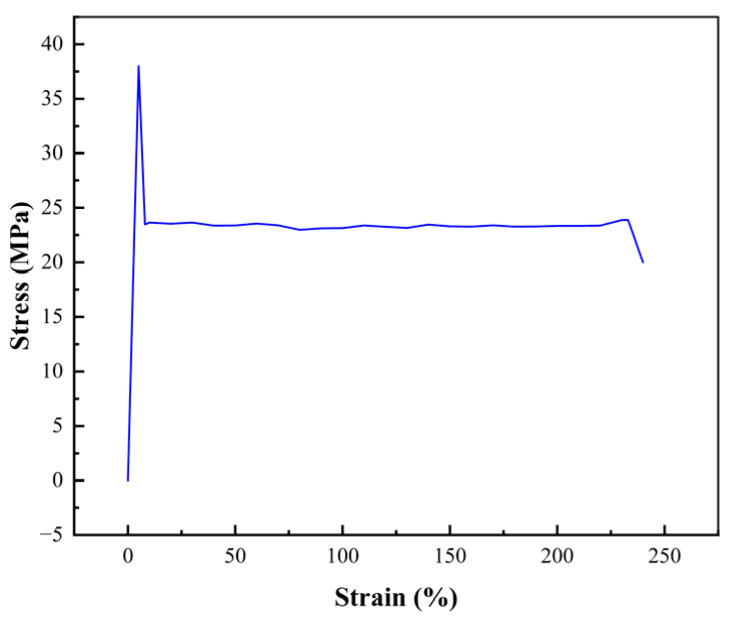
Stress–strain curves.

The stress-strain curve clearly illustrates the mechanical properties of the modified PVB film, as detailed in Figure 7 and Table 1. Compared with traditional PVB films, the modified film markedly increased the tensile strength, reaching 23.85 MPa. Upon the integration of the thermochromic powder, which is facilitated by weak hydrogen bonding, the elongation at break is elevated to 233.4%, surpassing the 200% elongation at break typical of conventional PVB. It is widely recognized that robust hydrogen bonds between PVB polymer chains can impede the processability and deformability of a film; however, the introduction of a modest amount of plasticizer is insufficient to disrupt these bonds [35].

Compared with its traditional counterpart, the modified PVB film has a diminished impact value of 7. This reduction in impact value is attributed to the subtle alteration in the hydrogen bond strength upon the addition of the thermochromic powder. The incorporation of this powder into the PVB matrix is posited to distribute tensile stress more effectively, thus enhancing elongation at break. This suggests that the modified thermochromic powder within the PVB contributes to a more ductile and resilient film, with improved mechanical properties that are particularly beneficial for applications requiring flexibility and impact resistance.

### 3.7. Outdoor Experimental Characterization

The analysis of the impact of three distinct types of external window glass on the indoor thermal environment has indicated that each glass variant significantly contributes to a reduction in the indoor temperature and an increase in the thermal comfort level. In terms of temperature control effectiveness, thermochromic dimming insulating glass emerged as the superior performer, with double silver low-emissivity (Low-E) insulating glass following runner-up and single silver low-E insulating glass taking the last position.

**Figure 8:**
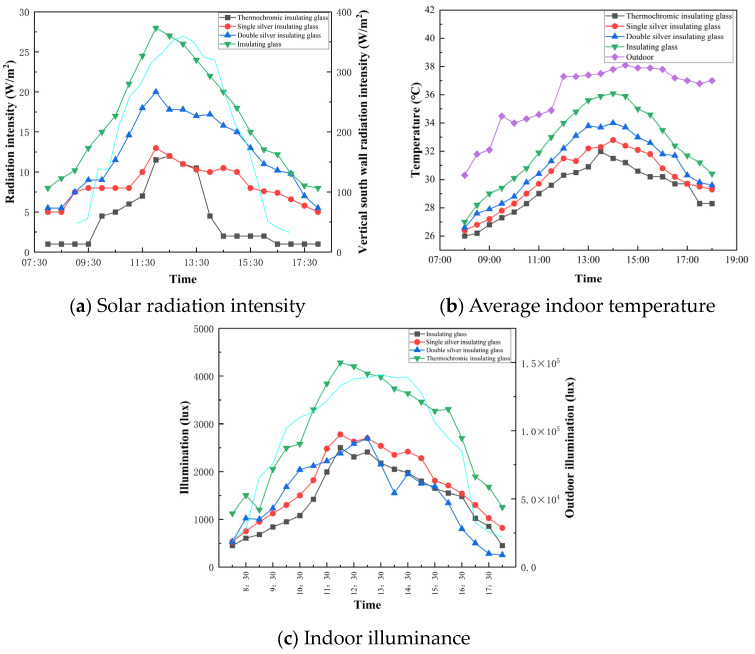
Southward comparison data under different outer windows in summer. The light blue line in (**a**) represents the irradiation intensity of the outdoor vertical facade, while the light blue line in (**c**) represents the outdoor illumination.

When examining the temperature differential across the glass surfaces, as shown in Figure 8, the single silver low-E insulating glass exhibited the smallest disparity between its inner and outer surfaces, which was lower than that of its double silver counterpart and substantially lower than the disparity observed with thermochromic dimming insulating glass. Notably, the inner surface temperature of the thermochromic dimming insulating glass remained the coolest, averaging 2.8 °C cooler than that of the single silver low-E insulating glass. This reduced surface temperature results in a decreased impact of thermal radiation on both objects and individuals within the room.

In terms of mitigating solar radiation intensity, the pivotal period requiring control in south-facing rooms is from 11:00 to 15:00, when solar radiation is at its zenith. The thermochromic dimming insulating glass demonstrated exceptional performance in controlling direct solar radiation through windows. This type of glass possesses the lowest solar radiation heat transfer coefficient, suggesting that its reduction in indoor solar radiation heat is primarily through managing the portion absorbed by the glass that is subsequently conveyed indoors as heat. Consequently, thermochromic dimming insulating glass significantly outperforms the other types of thermal insulation, with double silver low-E insulating glass ranking second and single silver low-E insulating glass being the least effective.

On the basis of data collected during a typical summer day, the indoor environment experiences continuous heating due to the combined effects of heat transfer from the indoor–outdoor temperature difference and solar radiation. With respect to the impact on the indoor lighting environment, thermochromic glazing ensures ample illumination even under overcast conditions during the testing period, owing to its 64% visible light transmittance. Consequently, compared with single- and double-silver low-E insulating glass, thermochromic glazing not only offers superior temperature control but also provides excellent lighting effects.

## 4. Conclusions

To succinctly encapsulate the research findings on the development of tunable thermochromic properties through emulsion polymerization and the newly developed application of thermochromic powders in smart film technology, the following key points encapsulate the outcomes of our rigorous experimental and investigative efforts:

(1) The incorporation of the ODA monomer in emulsion polymerization has been empirically demonstrated to produce a powder with thermally responsive discoloration properties. A specific concentration of 75% ODA within the polymer matrix facilitates a color change at 40.2 °C while maintaining over 90% visible light transmittance. Moreover, the transition temperature is adjustable within a precise range of 24.5 °C to 39.5 °C by modulating the ALMA content.
(2) Comprehensive analytical tests, including DSC-TG and SEM, confirmed the thermal characteristics and morphology of the synthesized powder. The powder comprises spherical particles with a remarkably uniform diameter of approximately 10 μm, which enhances its thermal processing capabilities and potential for effective anchoring within a matrix.
(3) Integrating thermochromic powder into insulating glass results in the creation of thermochromic smart laminated glass with adjustable shading capabilities, significantly enhancing thermal performance. Compared with single silver low-emissivity glass, this smart glass maintains the lowest inner surface temperature, with a notable temperature difference of 2.8 °C. This feature substantially reduces thermal radiation, thereby improving occupant comfort and protecting interior objects from excessive heat exposure. Furthermore, compared with double silver low-E glass, it achieves a 20–30% reduction in solar irradiance within the test rooms.
(4) In terms of indoor lighting conditions, thermochromic laminated glass offers effective control over direct sunlight. Its adjustable shading feature allows for the entry of diffused light, which can reduce the reliance on artificial illumination within the interior. By diminishing the need for artificial lighting or active cooling systems, this capability has the potential to lead to significant energy savings.

In the future, further testing is needed for thermochromic PVB, including plans for year-round performance assessments to evaluate the service life and aging characteristics under various environmental conditions. It is essential to verify the long-term stability and efficiency of our material, further optimize the formulation and synthesis process of the thermochromic materials and assess the full life cycle of the finished products. These innovations are expected to play a pivotal role in enhancing building energy efficiency and reducing the carbon footprint. Their applications are anticipated to extend across various sectors, including large-scale shading smart windows, daylight-responsive roofing systems, and glass greenhouses, thereby contributing to the advancement of sustainable architectural practices.

## Figures and Tables

**Table 1:** Tensile strength, elongation at break, and breakage rate of the samples.

Performance	Thickness (mm)	Tensile Stress (MPa)	Elongation at Break (%)	Melt Index (g/10 min)	Peel Force (N/mm)
Test value (1)	0.76	23.85	233.4	1.35	35
Test value (2)	0.75	23.83	233.1	1.35	35
Test value (3)	0.76	23.86	233.4	1.35	35

## Data Availability

Data are contained within the article.
